# P-459. Length Of Hospital Stay, In-patient Mortality And Factors Associated With Mortality Among *HIV-Cryptococcal Meningitis* Patients Receiving Liposomal Amphotericin B At Tertiary Hospitals in Uganda

**DOI:** 10.1093/ofid/ofae631.659

**Published:** 2025-01-29

**Authors:** Olivie Carolyne Namuju, Richard Kwizera, Lillian Tugume, David R Boulware, David Meya

**Affiliations:** Infectious Diseases Institute, Kampala, Kampala, Uganda; Infectious Diseases Institute Ltd, Mulago, Kampala, Uganda; Infectious Diseases Institute, Makerere University, kampala, Kampala, Uganda; University of Minnesota, Minneapolis, Minnesota; Infectious Diseases Institute, Makerere University, kampala, Kampala, Uganda

## Abstract

**Background:**

Cryptococcal meningitis (CM) remains the second lethal opportunistic infection among People Living with HIV (PLWHIV) in sub-Saharan Africa. Currently, the patients are managed on single high-dose liposomal amphotericin B and flucytosine. However, there is no sufficient data on whether the new treatment regimen has an effect on reducing inpatient mortality and length of hospital stay in the real-world setting.

We aimed to investigate the proportion of inpatient mortality, length of hospital stay (LOS), and factors associated with mortality among patients with HIV-associated CM receiving liposomal amphotericin B-flucytosine regimen.

**Methods:**

This was a cross-sectional study conducted to review medical records of patients admitted between December 2022 and May 2023 at 11 tertiary hospitals in Uganda. Medical records of 173 HIV-CM patients were reviewed. Univariate descriptive statistics were used to summarize the background characteristics. Modified Poisson regression was used to ascertain factors associated with mortality at bivariable and multivariable levels. Associations were presented through adjusted prevalence ratios with their 95% confidence intervals. Data were analyzed using STATA v15.

**Results:**

Of the 173 patients’ medical records reviewed, 58.4% were males with a median age of 38 years (IQR= 30, 48) and over 55.5% were married. Patients with altered mental status (GCS< 15) were 40%. Overall, inpatient mortality for liposomal amphotericin B was 35.8% and this varied by health facility. The median LOS was 7 days (IQR = 3, 12). Factors associated with mortality were convulsion [APR, 1.86, 95%CI (1.21-2.86), p-value=0.005)], altered mental status [APR; 1.66, 95%CI (1.07-2.57), p-value=0.023], and a comorbid condition [APR, 2.26 95%CI (1.44-3.53), p-value=0.006]. Therapeutic lumbar punctures were significantly associated with reduced mortality [APR; 0.47, 95%CI (0.29-0.73), p-value=0.001].

**Conclusion:**

Liposomal amphotericin B regimen has lower mortality than deoxycholate in the real-world setting. Patients on average spend more than a week in hospital. Male sex, convulsion, altered mental status, and comorbid conditions were independently associated with mortality. Therapeutic LPs significantly reduced inpatient mortality.

**Disclosures:**

**All Authors**: No reported disclosures

